# Development and Characterization of Novel Films Based on Sulfonamide-Chitosan Derivatives for Potential Wound Dressing

**DOI:** 10.3390/ijms161226204

**Published:** 2015-12-15

**Authors:** Oana Maria Dragostin, Sangram Keshari Samal, Florentina Lupascu, Andreea Pânzariu, Peter Dubruel, Dan Lupascu, Cristina Tuchilus, Cornelia Vasile, Lenuta Profire

**Affiliations:** 1 Department of Pharmaceutical Chemistry, Faculty of Pharmacy, “Grigore T. Popa” University of Medicine and Pharmacy Iasi, 16 University Street, Iasi 700115, Romania; oana.dragostin@umfiasi.ro (O.M.D.); florentina-geanina.l@umfiasi.ro (F.L.); andreea.panzariu@umfiasi.ro (A.P.); dan.lupascu@umfiasi.ro (D.L.); 2 Laboratory of General Biochemistry and Physical Pharmacy, Centre for Nano- and Biophotonics, Ghent University, Ottergemsesteenweg 460, Ghent 9000, Belgium; sksamalrec@gmail.com; 3 Polymer Chemistry & Biomaterials Research Group, Ghent University, Krijgslaan 281, S4-Bis, Ghent 9000, Belgium; peter.dubruel@ugent.be; 4 Department of Microbiology, Faculty of Pharmacy, “Grigore T. Popa” University of Medicine and Pharmacy Iasi, 16 University Street, Iasi 700115, Romania; cristina.tuchilus@umfiasi.ro; 5 Department of Physical Chemistry of Polymers, “Petru Poni” Institute of Macromolecular Chemistry, 41A Grigore Ghica Voda Alley, Iasi 700487, Romania; cvasile@icmpp.ro

**Keywords:** sulfonamide-chitosan derivatives, film, swelling ratio, biodegradation, antioxidant effects

## Abstract

The objective of this study was to develop new films based on chitosan functionalized with sulfonamide drugs (sulfametoxydiazine, sulfadiazine, sulfadimetho-xine, sulfamethoxazol, sulfamerazine, sulfizoxazol) in order to enhance the biological effects of chitosan. The morphology and physical properties of functionalized chitosan films as well the antioxidant effects of sulfonamide-chitosan derivatives were investigated. The chitosan-derivative films showed a rough surface and hydrophilic properties, which are very important features for their use as a wound dressing. The film based on chitosan-sulfisoxazol (CS-S6) showed the highest swelling ratio (197%) and the highest biodegradation rate (63.04%) in comparison to chitosan film for which the swelling ratio was 190% and biodegradation rate was only 10%. Referring to the antioxidant effects the most active was chitosan-sulfamerazine (CS-S5) which was 8.3 times more active than chitosan related to DPPH (1,1-diphenyl-2-picrylhydrazyl) radical scavenging ability. This compound showed also a good ferric reducing power and improved total antioxidant capacity.

## 1. Introduction

Chitosan is a natural, nontoxic, biocompatible and biodegradable polymer with a great variety of biomedical applications. This polymer is used in drug delivery, cell delivery systems, regenerative medicine, tissue engineering, *etc*. [1]. Chitosan also shows important biological effects such as antimicrobial, antioxidant, antidepressant, antitumor, hemostatic, anticholesteremic and antidiabetic effects that sustain its use as a biomaterial [2]. From the chemical point of view chitosan is a cationic polyelectrolyte at acidic pH with two types of reactive groups (amino and hydroxyl) that could be chemically modulated in order to improve its properties. By the functionalization of chitosan, new *N*-acylated, *N*-sulphated, *N*/*O*-carboxymethylated and *N*/*O*-substituted derivatives with increased biocompatibility, water solubility, and antioxidant and antimicrobial effects have been obtained [3]. For biomedical application chitosan was developed in different forms as films, gels, particles, membranes and scaffolds [4,5]. Similar to other polymers, chitosan has an excellent ability to form film, good mechanical strength and a high swelling degree [6]. The solubility of chitosan is closely related to the pH of the environment. The free amino groups of chitosan are protonated in acid solution, and the molecules become soluble. This solution can be used to form films by airdrying and porous scaffolds by freeze drying [7]. The use of unmodified chitosan to form films is limited due its high moisture permeability and brittleness [8].

To increase the antimicrobial properties of chitosan, new sulfonamide derivatives have been synthesized [9]. In a continuation of our previous work, in this paper we present the development and characterization of new films based on chitosan-sulfonamide derivatives with potential biomedical applications, especially as a wound dressing. The research showed that an ideal wound dressing should provide a moist environment with good biocompatibility and prevent bacterial infection to accelerate the tissue regeneration. Properties of films important to characterize an ideal wound dressing includin, morphology analysis, contact angle, swelling ratio and biodegradation were investigated. The swelling ratio influences the adsorption of exudates that are produced during the wound healing process while the biodegradation capacity is important for the use of dressings in treating wounds, without being absolutely required to have a regular replacement. Moreover, the biological performance of some biomaterials is linked to their degradation behavior whereas this ability influences cell performances and inflammatory response [10]. The major advantages of using this natural polymer as a wound dressing are its good cytocompatibility and biodegradability [11]. In addition, the antioxidant effects of chitosan-sulfonamide derivatives have been evaluated based on the implication of radical oxygen species in the wound healing process. Overproduction of free radicals is an important factor in wound chronicity, these being involved in normal aerobic metabolism, while their generation is relatively high during infections. This can be explained by the fact that free radicals constantly oxidize the new biomolecules produced in chronic wounds while acting as promoters of the pro-inflammatory response [12].

## 2. Results and Discussion

### 2.1. Chitosan Derivative Films

**Figure 1:**
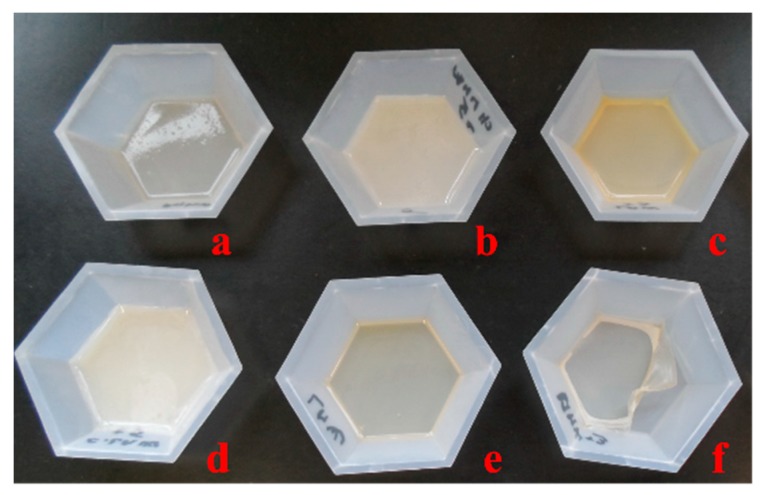
Macroscopic images of films: (**a**) CS-S1 (chitosan-sulfametoxydiazine); (**b**) CS-S2 (chitosan-sulfadiazine); (**c**) CS-S3 (chitosan-sulfadimethoxine); (**d**) CS-S4 (chitosan-sulfamethoxazol); (**e**) CS-S5 (chitosan-sulfamerazine); and (**f)** CS-S6 (chitosan-sulfizoxazol).

Chitosan (medium molecular weight) was functionalized with sulfonamide drugs (sulfametoxy-diazine, sulfadiazine, sulfadimethoxine, sulfamethoxazol, sulfamerazine, sulfizoxazol) in order to improve its biological properties, especially the antimicrobial activity. In a continuation of our research, starting with the sulfoanamide-chitosan derivatives, new films were developed. The morphology and the physical properties of chitosan films are presented. The resulting films had a thickness of approximately 0.1 mm and were colorless or slightly yellow (Figure 1).

### 2.2. Surface Morphology

**Figure 2:**
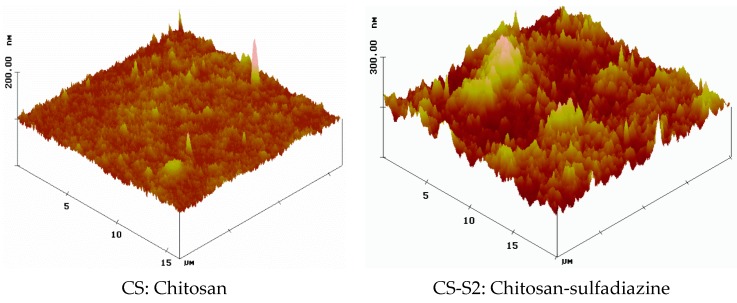
AFM image of chitosan (CS) and of chitosan-sulfadiazine (CS-S2) films.

The morphology of the dry films based on sulfoanamide-chitosan derivatives was studied using the Atomic Force Microscopy (AFM) technique which is used to observe the nano-scale features of the film surface. According to the AFM images, the chitosan-derivative films have a rougher surface compared to chitosan before functionalization, which is uniform and smooth. The three-dimensional (3D) images have clearly illustrated the increase of the number and height of the peaks for the chitosan-derivative film surfaces compared to the chitosan film surface. Figure 2 shows the AFM images of chitosan before functionalization (CS) and after functionalization using sulfadiazine (chitosan-sulfadiazine film CS-S2).

Many physico-chemical parameters of films such as adsorption, wettability, friction or electric contact properties are dependent on surface morphology [13]. In our case, considering all these aspects, the rough surface of the chitosan-derivative films will improve their adsorption capacity and consequently will increase their hydrophilicity. As these properties are improved, the adsorption capacity of the exudates in the wound healing process will be also increased.

### 2.3. Contact Angle Measurement

**Figure 3:**
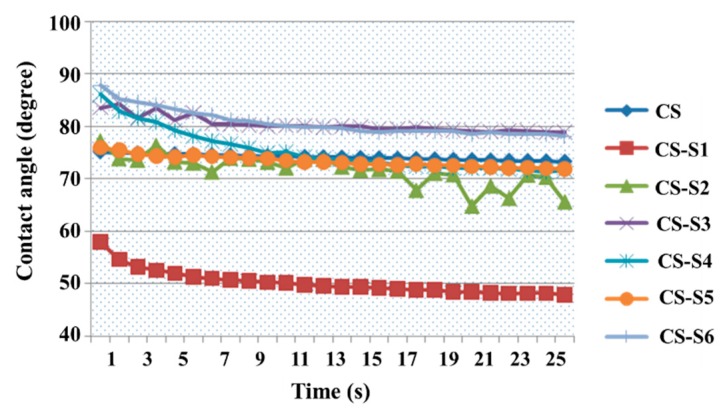
Water contact angle measurements for chitosan-derivative films.

The results obtained for chitosan-derivative films compared with chitosan are presented in Figure 3. The water contact angle value for chitosan film at moment zero is 75.06°, while the values for chitosan derivatives are between 57.98° (CS-S1) and 87.84° (CS-S6), depending on the structure of the sulfonamide used to functionalize the chitosan. Even if, for some chitosan derivatives (CS-S3, CS-S6), the value of the water contact angle recorded over 30 s was easily increased compared to chitosan, it can be appreciated that the chitosan derivatives remain hydrophilic because the value of the water contact angle is less than 90°. The most hydrophilic compound is chitosan-sulfamethoxydiazine (CS-S1) with a contact angle value of 57.98° at time 0, and 47.81° at the end of the experiment.

**Figure 4:**
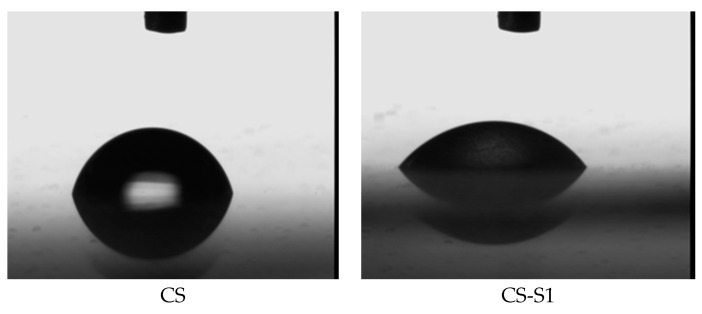
Images obtained during the measurements (time zero): chitosan (CS) and CS-sulfamethoxydiazine (CS-S1) films.

For exemplification, Figure 4 shows the images obtained for chitosan (CS) and chitosan-sulfamethoxydiazine (CS-S1) films, at moment zero of the experiment.

The lower water contact angles for most of the chitosan-derivative films can be explained by the sulfonamide structure. The polar character of the sulfonamide such as the methoxy group decreases the contact angle values of the corresponding films, which means increasing their hydrophilicity. In addition, the hydrophilicity is closely correlated with the substitution degrees of chitosan derivatives [14]. If the degree of substitution with polar structures is greater, the contact angle value is smaller, as is the case of chitosan-sulfamethoxydiazine (CS-S1, Figure 4). The opposite of this fact is found in the case of chitosan-sulfizoxazol when the sulfonamidic moiety has two methyl non-polar groups; in this case, the contact angle value increases with the decrease of its hydrophilicity.

### 2.4. The Mechanical Properties of Films

**Figure 5:**
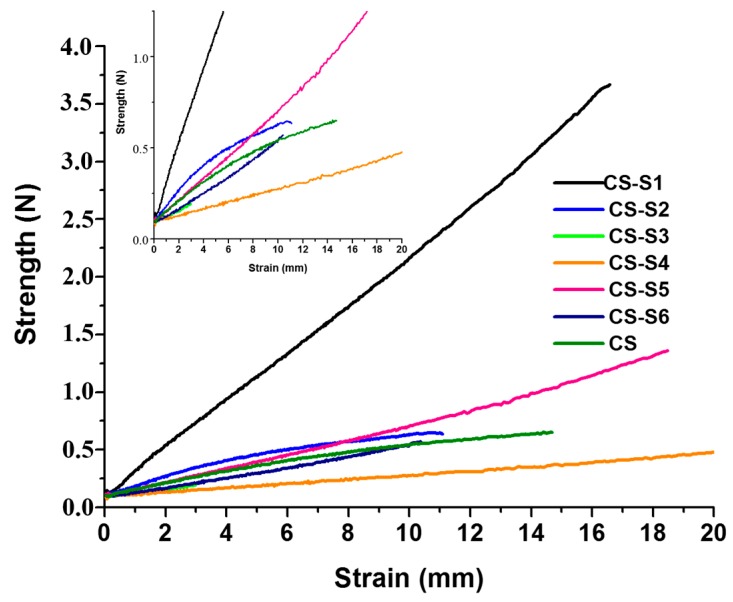
The strain (mm) of chitosan (CS) and chitosan-derivative films.

The strains (mm) at the breaks of chitosan (CS) and chitosan-derivative films are presented in Figure 5.

The strain at the break for chitosan film was 14.68 mm, recorded at value of 0.65 newtons (N). For almost all chitosan-derivative films the mechanical properties increase in comparison with the chitosan film. Among the chitosan derivatives, good mechanical properties were shown by CS-S1 (chitosan-sulfamethoxydiazine), CS-S5 (chitosan-sulfamerazine) and CS-S4 (chitosan-sulfamethoxazol) for which the strain at the break was 16.58 mm at 3.67 N, 18.48 mm at 1.36 N and 28.16 mm at 0.72 N, respectively.

### 2.5. Swelling Ratio Measurement

**Figure 6:**
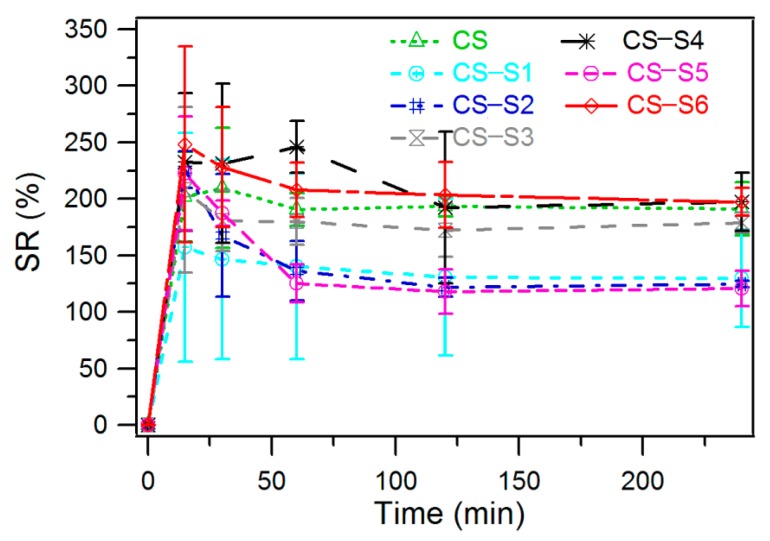
Swelling ratio of chitosan-derivative films.

For the chitosan film the thermodynamic equilibrium was achieved after 60 min when the recorded swelling ratio was 190% (Figure 6). Chitosan-derivative films showed a similar swelling ratio except for chitosan-sulfadiazine (CS-S2), chitosan-sulfamerazine (CS-S5) and chitosan-sulfamethoxydiazine (CS-S1). For these chitosan derivatives the thermodynamic equilibrium was achieved after 120 min (CS-S1, CS-S2) and 60 min (CS-S5), respectively, with a corresponding swelling ratio of 122%, 125% and 130%. The highest swelling ratio was recorded for chitosan-sulfisoxazol (CS-S6), for which the swelling ratio was 197% at thermodynamic equilibrium which was reached after 240 min.

### 2.6. In Vitro Biodegradation

Lysozyme is an enzyme that is present in exudate, tears, nasal secretions, saliva, urine and other body fluids. Exudate is generated during the wound healing process as part of the inflammatory response, and it is one of the components of the reparative process. Exudate has a complex composition that includes glucose, cytokines, leucocytes, lysozyme and many others components. Lysozyme is able to destroy certain bacteria, working by splitting the components of the extracellular matrix [15]. Moreover, *in vitro* biodegradation of chitosan and its derivatives is mainly attributed to the effect of lysozyme through the hydrolysis of acetylated residues. The degradation products of chitosan and its derivatives are d-glucosamine and glycosaminoglycan, which are non-toxic for mammalian cells [16]. Based on this idea, we chose to determine the ability of biodegradation of chitosan-derivative films in a phosphate buffered saline (PBS) solution (pH 7.4) containing lysozyme at 37 °C.

**Figure 7:**
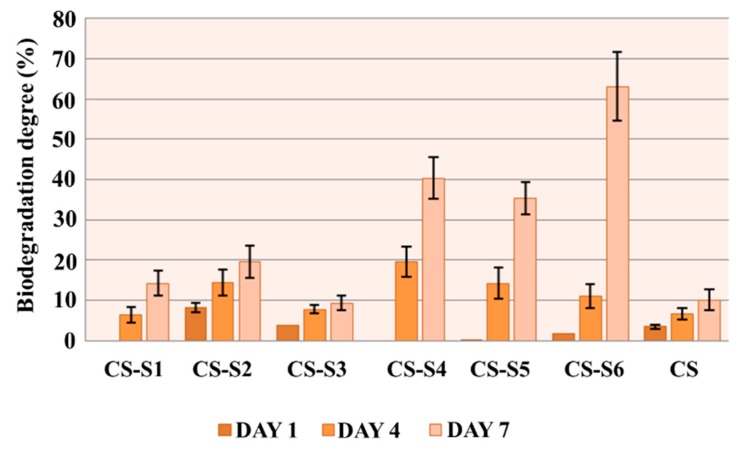
*In vitro* biodegradation of chitosan-derivative films.

The results of the *in vitro* biodegradation of chitosan-derivative films are presented in Figure 7. With the exception of the chitosan-sulfamethoxydiazine (CS-S1) and chitosan-sulfadimethoxine (CS-S4) films, for which no biodegradation was recorded on the first day of the experiment, the rest of the compounds showed biodegradation on the first day and it increased during the experiment.

At the end of the experiment, all chitosan-derivative films were biodegraded at a higher ratio than chitosan, for which the biodegradation ratio was 10%. The highest rate of biodegradation was recorded for the chitosan-sulfizoxazole film (CS-S6), which was biodegraded at a ratio of 63.04% on the seventh day of the experiment. This could be explained by the rough surface of the chitosan-derivative films, which assures a greater contact surface with the biological medium containing lysozyme.

### 2.7. Antioxidant Assays

#### 2.7.1. Total Antioxidant Capacity

**Figure 8:**
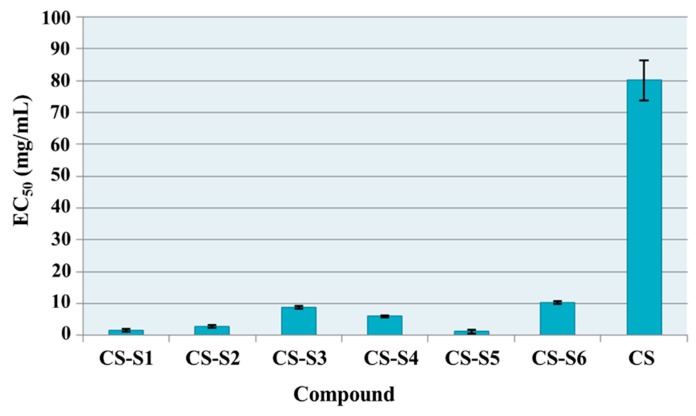
Total antioxidant capacity (EC_50_ mg/mL) of chitosan derivatives.

The effective concentrations (EC_50_) for chitosan derivatives in comparison with chitosan are presented in Figure 8. All chitosan derivatives showed higher total antioxidant capacity than chitosan. For chitosan the EC_50_ value was 80.11 mg/mL while chitosan derivatives have EC_50_ values between 10.28 and 1.14 mg/mL. The most active derivatives were chitosan-sulfadiazine (CS-S2, EC_50_ = 2.74 mg/mL), chitosan-sulfamethoxydiazine (CS-S1, EC_50_ = 1.61 mg/mL) and chitosan-sulfamerazine (CS-S5, EC_50_ = 1.14 mg/mL). These derivatives were 29 times (CS-S2), 50 times (CS-S1) and 70 times (CS-S5) respectively more active than chitosan (CS).

#### 2.7.2. Reducing Power

**Figure 9:**
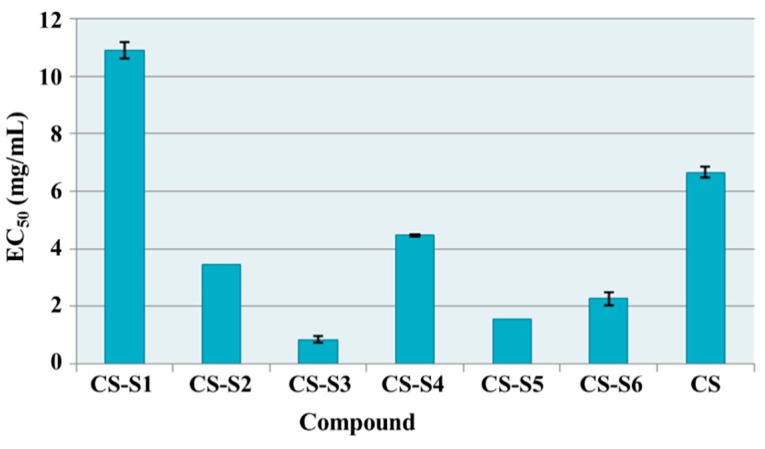
Ferric reducing power (EC_50_ mg/mL) of chitosan derivatives.

The reducing power of chitosan derivatives was evaluated based on the reduction of the ferric/ferricyanide complex to its ferrous (Fe^2+^) state known as Prussian blue dye. It was observed that chitosan derivatives, except chitosan-sulfamethoxydiazine (CS-S1, EC_50_ = 10.92 mg/mL), have more intense reducing power than chitosan (EC_50_ = 6.65 mg/mL) (Figure 9). The most active were chitosan-sulfadimethoxine (CS-S3) and chitosan-sulfamerazine (CS-S5). For these compounds the value of EC_50_ was 0.85 mg/mL (CS-S3) and 1.54 mg/mL (CS-S5) which means they are 7.7 times and 4.3 times respectively more active than chitosan. Good activity was shown also by chitosan-sulfizoxazol (CS-S6, EC_50_ = 2.21 mg/mL), chitosan-sulfadiazine (CS-S2, EC_50_ = 3.54 mg/mL) and chitosan-sulfamethoxazol (CS-S4, EC_50_ = 4.62 mg/mL).

#### 2.7.3. DPPH (1,1-diphenyl-2-picrylhydrazyl) Radical Scavenging Ability

DPPH is an organic radical, commonly used to evaluate the radical scavenging ability of antioxidants, which presents a maximum absorption band at about 517 nm. In the presence of the antioxidants it becomes colorless or pale yellow through the donation of a proton forming the reduced DPPH.

**Figure 10:**
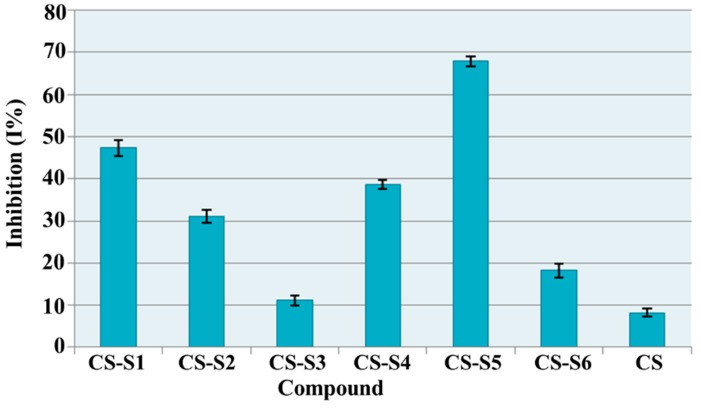
DPPH (1,1-diphenyl-2-picrylhydrazyl) radical scavenging ability (I %) of chitosan derivatives.

According to the obtained results (Figure 10), chitosan derivatives showed a good scavenging ability, which ranged between 11.16% (CS-S3) and 67.89% (CS-S5), while for chitosan (CS) this value was 8.14%. The most active compounds were chitosan-sulfamethoxydiazine (CS-S1, I% = 47.31%) and chitosan-sulfamerazine (CS-S5, I% = 67.89%). These compounds are 5.8 times and 8.3 times respectively more active than chitosan.

### 2.8. Antimicrobial Assays

In this study sulfonamide-chitosan derivatives were prepared and their substitution degree was between 9.61% and 34.24%. The antimicrobial activity was manifested at the sample surface in direct contact with bacterial media.

#### Diameter of Inhibition Area

Chitosan derivatives which include, in the same structure, two different entities with a known antimicrobial action, chitosan and sulfonamide, could have increased antimicrobial activity. In this context, the chitosan-derivative films were assayed for their antimicrobial effects against five different bacterial strains (*Staphloccocus auresus* ATCC 25923, *Sarcina lutea* ATCC 9341, *Bacillus cereus* ATCC 14579, *Bacillus subtilis,* *Escherichia coli* ATCC 25922) and three fungal strains (*Candida albicans* ATCC 10231, *Candida glabrata* ATCC MYA 2950, *Candida sake*). The antimicrobial activity of chitosan-derivative films compared to the chitosan film (CS), expressed as diameters of inhibition area (mm), is presented in Table 1. Nitrofurantoine and nystatine were used as reference drugs (Table 2).

**Table 1:** Diameter of inhibition area (mm) of chitosan film (CS) and chitosan-derivative films.

Film	Diameter of Inhibition Area (mm)							
	*S.a.*	*S.l.*	*B.c.*	*B.s.*	*E.c.*	*C.a.*	*C.g.*	*C.s.*
**CS**	16	28	20	20	15	14	0	0
**CS-S1**	15	30	22	18	19	10	12	17
**CS-S2**	17	30	27	25	28	20	25	32
**CS-S3**	18	30	15	20	19	11	0	0
**CS-S4**	17	30	27	22	22	0	25	32
**CS-S5**	15	30	20	18	17	0	0	0
**CS-S6**	17	28	16	18	18	0	0	9

*S.a.—Staphloccocus auresus* ATCC 25923; *S.l.—Sarcina lutea* ATCC 9341; *B.c.—Bacillus cereus* ATCC 14579; *B.s.—Bacillus subtilis*; *E.c.—Escherichia coli* ATCC 25922; *C.a.—Candida albicans* ATCC 10231; *C.g.—Candida glabrata* ATCC MYA 2950; *C.s.—Candida sake*.

**Table 2:** Diameter of inhibition area (mm) of nitrofurantoine and nystatine.

Control	Diameter of Inhibition Area (mm)							
	*S.a.*	*S.l.*	*B.c.*	*B.s.*	*E.c.*	*C.a.*	*C.g.*	*C.s.*
Nitrofurantoine 300 µg/disc	19	8	12	20	20	n.d.	n.d.	n.d.
Nystatine 100 µg/disc	n.d.	n.d.	n.d.	n.d.	n.d.	29	26	31

*S.a.—Staphloccocus auresus* ATCC 25923; *S.l.—Sarcina lutea* ATCC 9341; *B.c.—Bacillus cereus* ATCC 14579; *B.s.—Bacillus subtilis*; *E.c.—Escherichia coli* ATCC 25922; *P.a.—Pseudomonas aeruginosa* CIP 82118; *C.a.—Candida albicans* ATCC 10231; *C.g.—Candida glabrata* ATCC MYA 2950; *C.s.—Candida sake*; n.d.—not determined.

As shown in Table 1, all tested films have a good antimicrobial activity against all bacterial strains, their activity being comparable or more intense than chitosan, depending on the bacterial strain. The results support the good influence of the sulfonamide structure on the antibacterial activity of chitosan, especially in the case of the CS-S2 and CS-S4 films. Compared with nitrofurantoine, used as a reference drug, all tested films have a similar or even better activity. Against fungal strains, only CS-S1, CS-S2 and CS-S4 were active, the activity of CS-S2 and CS-S4 being comparable with nystatine.

Cell proliferation tests of chitosan-sulfoanamide derivatives using mouse fibroblasts have already been done and the results were included in a previous paper [14]. It was found that all samples are biocompatible and do not show cytotoxicity.

## 3. Experimental Section

### 3.1. Materials

Chitosan medium molecular weight (CS, 425 kDa, deacetylation degree of 85%), acetic acid, sodium tripolyphosphate (TPP), sodium phosphate, ammonium molybdate, potassium ferricyanide, trichloroacetic acid, ferric chloride, 1,1-diphenyl-2-picrylhydrazyl (DPPH) were purchased from Sigma Aldrich Company (Taufkirchen, Germany). Lysozyme with an activity of 10,000 UI/Ml was obtained from Merck Company (Darmstadt, Germany). The chitosan derivatives used were: used chitosan-sulfametoxydiazine, chitosan-sulfadiazine, chitosan-sulfadimethoxine, chitosan-sulfamethoxazol, chitosan sulfamerazine and chitosan-sulfizoxazol. All solvents and chemicals had a high degree of purity.

### 3.2. Preparation of Chitosan Derivatives Films

Chitosan-derivative films were prepared using new chitosan-sulfonamide derivatives (CS-S1, CS-S2, CS-S3, CS-S4, CS-S5, CS-S6). The synthesis of new chitosan-sulfonamide derivatives was performed according to reference [9]. Briefly, by reaction of sulfonamides (sulfametoxydiazine, sulfadiazine, sulfadimethoxine, sulfamethoxazol, sulfamerazine, sulfizoxazol) with chloroacetyl chloride, the corresponding chloroacetyl-sulfonamide derivatives were obtained. Further, the chloroacetyl-sulfonamides were reacted with chitosan so that new chitosan-sulfonamide derivatives were obtained.

In order to prepare the films, the chitosan medium molecular weight (CS) and chitosan-sulfonamide derivatives (CS-S1, CS-S2, CS-S3, CS-S4, CS-S5, CS-S6) in a concentration of 2% (*w*/*v*) were dissolved by stirring in acetic acid solution 2%. A volume of 20 mL of obtained solutions was poured into the polystyrene Petri plates (10 cm × 10 cm) and then the plates were kept at room temperature for 48 h. The films were crosslinked with a solution of 5% sodium tripolyphosphate (TPP), washed several times with water, in order to remove all the acetic acid and TPP, and dried at room temperature for one week according to the literature data [17,18,19].

The thickness of films has been measured using a digital micrometer (Mitutoyo High-Accuracy Digimatic Micrometer (Kruibeke, Belgium) with 1 µm accuracy. For each film three measurements in different positions were made.

### 3.3. Surface Morphology

The surface morphology of films was analyzed using a Multimode Scanning Probe Microscope (Digital Instruments, Milano, Italy) equipped with a Nanoscope IIIa controller. The 15 mm scans are recorded in tapping mode with a silicon cantilever (OTESPA, Veeco, Bruker, Billerica, MA, USA). The recorded images were modified with an X and Y Plane Fit Auto procedure and the surface roughness was analyzed using Nanoscope software version 4.43r8 (Digital Instruments, Milano, Italy).

### 3.4. Contact Angle Measurement

The contact angle for chitosan-derivative films was determined by the sessile drop method using the OCA 20 System (DataPhysics Instrument GmbH, Filderstadt, Germany, distributed by Benelux Instrument). To control the drop size of the water the system includes a high precision liquid dispenser. On the surface of chitosan-derivative film, 1 µL of water was placed using a Hamilton syringe. The shape of droplet was designed on the screen. A CCD video camera using 1 frame/s was used to record the image of water droplet [20]. Subsequently, the static contact angle was determined based upon the Laplace Young fitting using the imaging software provided by the supplier (SCA 20, version 2.1.5 build 16, DataPhysics Instrument GmbH, Filderstadt, Germany). The contact angle formed between the pure liquid and solid surface over the course of 30 s was measured and its value at every second was recorded. The experiment was performed in triplicate.

### 3.5. The Mechanical Properties

The strain of the films in wet state was measured by using a universal testing system (model 5564, Instron Corporation, Canton, MA, USA) according to the procedure based on ASTM Standard D1708, at room temperature [21]. Chitosan and chitosan-derivatives film, after being well moisturized with a 0.1 M PBS solution, were cut into 2.5 cm × 0.4 cm pieces and then were mounted on the two-grip handle and with a constant speed of 1 mm/min, under 65% relative humidity, were pulled to break.

### 3.6. Swelling Ratio

The swelling behavior of the crosslinked chitosan-derivative films was studied by incubation at 37 °C, to assess the hydrophilic character of the chitosan derivatives. The films were cut into small pieces with equal weights (*W*_d_) and then were introduced in double-distilled water. At different times the film sample was taken out, the excess of water was removed using filter paper and weighted (*W*_w_) and then placed again in water. The swelling ratio (SR) was calculated using the following formula:

SR (%) = (*W*_w_ − *W*_d_)/*W*_d_ × 100 (1)

where *W*_d_ and *W*_w_ are initial mass and mass at different time intervals, respectively [15].

### 3.7. In Vitro Biodegradation

The biodegradation of chitosan-derivative films was studied in phosphate buffered saline (PBS) (pH 7.4) containing lysozyme at 37 °C. Film samples with equal weight were immersed in PBS solution until the swelling equilibrium was attended, then the PBS solution was changed with the PBS solution containing lysozyme (10,000 UI/mL) and incubated at 37 °C for seven days. At different times (one day, four days, seven days) the film sample was taken out and weighed. The biodegradation degree was determined by using the next formula:

*D* (%) = (*W*_0_ − *W*_x_)/*W*_0_ × 100 (2)

where *W*_0_ and *W*_X_ are the wet weight before incubation and after incubation, respectively [22].

### 3.8. Antioxidant Assays

The antioxidant effects were assessed through *in vitro* tests: total antioxidant capacity, ferric reducing power and radical scavenging ability, by using a UV-VIS Spectrophotometer: CINTRA 2020 (GBC Scientific Equipment, Braeside, Melbourne, Australia).

#### 3.8.1. Total Antioxidant Capacity

The antioxidant capacity of chitosan derivatives was assessed based on phosphomolybdenum method [23]. To 50 µL of stock solution (5 mg/mL in 2% acetic acid) was added 2 mL of reagent solution (sulphuric acid 0.6 M, sodium phosphate 28 mM and ammonium molybdate 4 mM). The sample was incubated for 90 min at 95 °C and then was cooled at room temperature. The absorbance at 695 nm was measured using a blank that contain 50 µL of 2% acetic acid and 2 mL of reagent solution. Effective concentration (EC_50_) for each chitosan derivative was calculated. The experiment was performed in triplicate.

#### 3.8.2. Ferric Reducing Power

The ferric reducing power of the chitosan derivatives was assessed based on method described in the literature [24] with minor modifications. To 1 mL of stock solution (5 mg/mL in 2% acetic acid), was added 1 mL of sodium phosphate buffer (0.2 M, pH = 6.6) and 1 mL of potassium ferricyanide (1% *w*/*v*). The sample was incubated for 20 min at 50 °C. After that, 1 mL of trichloroacetic acid (10% *w*/*v*) was added to stop the reaction. The sample was centrifuged at 4500 rpm for 15 min. The upper layer (1 mL) was diluted with 1 mL of deionized water and then 0.2 mL of ferric chloride (0.1% *w*/*v*) was added. The absorbance at 700 nm was measured after 5 min, using a blank (that contain all reagents less stock solution that was replaced with 1 mL of acetic acid 2%). Effective concentration (EC_50_) for each chitosan derivative was calculated. The experiment was performed in triplicate.

#### 3.8.3. DPPH (1,1-diphenyl-2-picrylhydrazyl) Radical Scavenging Ability

The radical scavenging ability of the chitosan derivatives against 1,1-diphenyl-2-picrylhydrazyl (DPPH) was assessed based on the method described in literature [25,26,27] with minor modifications. To 50 µL of stock solution (20 mg/mL in 2% acetic acid) was added 2950 µL of DPPH solution (0.1 nM in methanol). The sample was left for 30 min at room temperature in the dark, and then the absorbance at 517 nm was measured. As control DPPH methanol solution was used. The radical scavenging ability was calculated using the following formula:

% inhibition (I%) = (*A*_c_ − *A*_s_)/*A*_c_ × 100 (3)

where *A*_c_ and *A*_s_ are the absorbance of the control and sample, respectively. The experiment was performed in triplicate.

### 3.9. Antimicrobial Assays

#### 3.9.1. Diameter of Inhibition Area

The antibacterial activity of the developed films was evaluated using agar disc diffusion method according to the method described in the literature [28]. Five bacterial strains: *Staphyloccoccus aureus* ATCC 25923, *Sarcina lutea* ATCC 9341, *Bacillus cereus* ATCC 14579, *Bacillus subtilis*, *Escherichia coli* ATCC 25922 and two yeast strains: *Candida albicans* ATCC 10231, *Candida glabrata* and *Candida sake* have been included in the study. All strains belong to the Culture Collection of the Department of Microbiology, Faculty of Pharmacy, “Gr. T. Popa” University of Medicine and Pharmacy, Iasi, Romania.

Each microbial culture was diluted in sterile 0.9% NaCl to obtain a turbidity of 10^6^ CFU/mL, according to the McFarland standard no. 0.5, which in the next step was diluted in Mueller Hinton agar (for bacteria) and Sabouraud agar (for yeasts) in the ratio of 1:10. The microbial medium was spread on sterile Petri plates (25 mL/Petri plate) and then sterile stainless steel cylinders (50 mm internal diameter; 100 mm height) were applied on the surface. Then 0.2 mL of samples (20 mg/mL in 1% acetic acid) (CS, CS-S1, CS-S2, CS-S3, CS-S4, CS-S5, CS-S6) was added to each cylinder. The Petri plates were incubated at 37 °C for 24 h (for bacteria) and at 24 °C for 48 h (for yeasts), and after that the diameter of inhibition area was measured. Commercially available discs containing nitrofurantoine (300 μg/disc) and ciprofloxacine (5 μg/disc) were used as positive control.

### 3.10. Statistical Analysis

All assays were carried out in triplicate. Data were analyzed by an analysis of variance (ANOVA) (*p* < 0.05) and were expressed as means ± SD. The total antioxidant activity (EC_50_ values) were calculated by linear interpolation between values above and below 50% activity.

## 4. Conclusions

New films based on six chitosan-sulfonamide derivatives (CS-sulfametoxydiazine, CS-sulfadiazine, CS-sulfadimethoxine, CS-sulfamethoxazol, CS-sulfamerazine, CS-sulfizoxazol) were developed and characterized. The films showed a rough surface and a hydrophilic character compared to chitosan film. For some of the chitosan-derivative films (CS-sulfamethoxydiazine, CS-sulfamethoxazol, CS-sulfizoxazol) the swelling ratio was similar and even higher than that of chitosan film. Also, based on the rough surface, all films of chitosan derivatives were biodegraded at a higher ratio than chitosan, for which the biodegradation ratio was only 10%. The sulfonamide-chitosan derivatives showed antioxidant effects compared with chitosan proved by *in vitro* assays (total antioxidant capacity, ferric reducing power, DPPH radical scavenging ability). The antimicrobial effects of chitosan-sulfonamide derivative films were also evaluated. The tested films, especially chitosan-sulfadiazine (CS-S2) and chitosan-sulfamethoxazol (CS-S4), showed good antibacterial and antifungal activity against all tested strains. Moreover, their antimicrobial activity was similar or even better than that of the reference substances nitrofurantoine and nystatine, respectively.

According to the results, the most effective films for biomedical applications such as wound-dressing materials are chitosan-sulfadiazine (CS-S2) and chitosan-sulfamethoxazol (CS-S4), which showed good antimicrobial and antioxidant effects. Moreover, these compounds are hydrophilic, with a good swelling degree and improved biodegradation rate compared to chitosan, which are also characteristics that are required for a good dressing material. In addition, the chitosan-sulfamethoxazol also showed good mechanical properties, which are also important for potential wound-dressing applications.

However, even progress has been made in the healing process, the control of infections, free radicals and inflammation remains a huge area of interest for researchers.
